# Valedictory Address to the Graduates of the Ohio College of Dental Surgery, of the Session of 1855–6

**Published:** 1856-04

**Authors:** J. Taft


**VALEDICTORY ADDRESS TO THE GRADUATES OF THE OHIO COLLEGE OF DENTAL SURGERY, OF THE SESSION OF 1855-6. BY PROF. J. TAFT.**

Man is by nature a social being. The hermit in his solitude, is looked upon by all as a being who violates laws, most obviously and indelibly impressed upon him.

This we assume: that mankind are placed in this world with a view to social intercourse. Out of this social relationship, duties and responsibilities arise. These are numerous and various in their character. Some of them are absolute in their nature; others are relative. Of the former we have an example in the relationship of parent and child, husband and wife. Other relations are subject to a greater range of vicissitudes.

There are several methods of reckoning the duties and responsibilities that devolve upon us as social beings. We may be able to determine something in regard to them, by considering the circumstances by which we are surrounded ; the period of the world in which we are made actors ; the light by which we are encompassed, and the locality in which our lot has been cast. By these and other things, let us discern if we can, the part that devolves upon us. The course of action we should pursue cannot rationally be determined independent of these surroundings. I propose then to notice some of the more obvious indications and tendencies of the age in which we live, with a view of ascertaining to some slight extent our duties and responsibilities.

A distinguishing characteristic of this age, is *increasing* and *advancing light.*

The 'present is marked by improvements, discoveries and elucidations of physical and moral truths hitherto unknown. In religion, science and politics, a flood of light has poured in upon the world. This, however, is of recent date.

"In the beginning darkness was upon the face of the deep."

Not less sad and gloomy was the darkness that enveloped the world in a moral and scientific aspect long after the beginning. The past history of the world exhibits but too plainly the gloom that pervaded it from near the beginning to almost the present period.

It was a darkness that could be felt, but could not be seen, for it enveloped men like the mist of mid-night. It penetrated the hall of science, and the schools of phylosophy. It shrouded the palaces of kings ; threw its dark pall over the chambers of legislation, and overlaid with its broad mantle almost the whole world. Here and there, at long intervals, the human mind cast forth a ray of light, but it was fleeting and gone. Occasionally an ephemeral light, meteor-like, would move, dance, and dazzle in the deep surrounding night, which, while it exhibited its own glory,served only to render the succeeding gloom more intense and appalling.

A change has taken place. That condition of things is now attained when knowledge shall be extended with an increased ratio. Whenever a new light springs up, it is supported and nourished, grows, and expands till all feel the genial influence.

In the dark ages, the lights rose and made a rapid passage to their culmination ; then swift declining sank beneath a horizon of impenetrable darkness. Now they seem to come from regions of day, and rise to their positions in the heavens, and there remain to cast forth their benignant rays, not only for the present, but give us an earnest for all coming time. Our knowledge of the world has extended farther in the last half century, than in five thousand years before. Till within the last few centuries our knowledge of the world is confined to a comparatively narrow belt of territory around the Mediterranean Sea. Now, there is no corner of earth so remote, no point so secluded, that we have not some acquaintance with it.

The geography, the physical characteristics and peculiarities, the manners and customs, and even the past history, so far as it may be gathered from traditionary relics and hieroglyphics, are all quite familiar to us. Many of the heretofore hidden parts of earth are so lucidly and strikingly described, that they seem as familiar as our own fire-sides. The time spoken of by the Prophet, "when knowledge shall be increased," we are permitted to see. We have but to make a glance to perceive this,—aye, it is almost impossible to avoid seeing it.

Science, reason and revelation have commingled their rays; and their concurrent testimony may be advanced to establish almost every truth. Science has become boundless ; she has pushed her researches everywhere. Vast and boundless are the recent extensions of those sciences the existence of which has been recognized from remote periods. And every day reveals some new science ; the expansion and full elucidation of which remains for the future.

In the full and boundless scope given to the human intellect we have assurance of grand discoveries yet to be made. The improvements made upon discoveries are usually of vastly more importance than the original discovery itself. Improvements in the arts and sciences are most rapidly made when there is the greatest freedom of thought. Scientific research extends everywhere. It examines the machinery of the universe, investigating its movements, and the laws by which it is governed. It is brought to bear upon all the objects of nature; none of such magnitude that it will not grapple with them; and none so small that it cannot apprehend them. The internal structure of the earth is opened up and spread out, and elucidated, so that it may be comprehended by the most ordinary capacity. A whole world of material beings that are invisible to the unassisted eye, are brought, by the aid of the microscope, under the most scrutinizing examination, even of all their various parts.

A rapid and general diffusion of knowledge is a distinguishing feature of the times. Whatever the schools of learning may have done for a few individuals in former times, they failed to give education to the masses of the people, so that they grew up and passed through life with little or no mental cultivation. A change has come over mankind. The disposition now is, in many parts of the world, at least, to educate all the people—all classes and ranks; and the time will soon arrive when the aim will be to educate every individual to the utmost capacity. The means of education have been multiplied a thousand fold, and though so much *has* been done, more is now being accomplished than at any former time. The schools and colleges, the halls of science, the studios and laboratories of art, all teem with life and activity. The cause of education has of late been receiving unwonted attention throughout the world. Nations and States have come to regard this as no inconsiderable part of their appropriate work.

The true nature of education, which consists in unfolding and directing aright the physical, intellectual and moral, social and religious natures, is beginning to be understood and appreciated. Its progressive nature is more commonly recognized; a mistaken notion upon this point has prevailed very extensively.

"Many who have had what was considered an education, have no idea of an education progressive through life. Having attained a certain measure of accomplishments, knowledge, manners, &c., they consider themselves made up, and so take their station. They are pictures which being quite finished are put in a frame, a gilded one if possible, and hung up in permanence of beauty, at least till old time with his rude and dirty fingers soils the charming colors."

Books are being multiplied to an endless extent. Works in almost every department of human knowledge are now within the reach of all. The press, scientific, political and religious, is scattering its sheets like leaves of autumn on every wind and distributing them to almost every habitation. In view of these facts, it requires not a Prophet's ken to discern the superior advantages that are flowing down to future generations through these channels. The present will not go to the future as the past has come to us. The past history of the world is a great scroll, with here and there an inscription upon it, and some of these scarcely legible; the present will go to the future, a roll closely written and on both sides.

The development and propagation of light and knowledge is fast becoming the ruling idea of the world. The angel of light is hovering over this once benighted world; beams of light radiating from his wings dispel the gloom that brooded on it; and by his might breaks in sunder, and causes to fall powerless, the fetters that bound mankind in ignorance, darkness and superstition.

The tendency of the times is to free and independent thought. Men are beginning to think and reason for themselves. In former periods of the world a few men thought for the whole race. The kind of knowledge entertained seemed to render this almost inevitable. Men were wont to place implicit faith and confidence in the declarations and reasonings of those they esteemed as their superiors. The time has passed away when men will base their faith upon the mere assertions and sophistical reasonings of others, of whatever station. No new theory or doctrine can be advanced, but that all will pass judgment upon it, and every one will arrive at his own conclusions, from his own reasonings; or at least the reasoning of others must find a verification in his own. A great majority of men, in the exercise of their thinking powers have remained in a state of perpetual infancy. On great truths they follow the consciences and opinions of reputed great men, and thus many who were moved along in the right direction by this extraneous force, obtained credit for that which had no existence.

"But a mind exhibiting such servility as to receive even truth and tenaciously adhere to it, on the simple authority of another, or from mere sympathy with others, is even more pitiable and contemptible, than one that in self reliance receives and defends error." To such minds in fact, there can be no real distinction between truth and error, as they are just as liable to entertain the one as the other, as sympathy, interest or authority may dictate. In all this a change is taking place. Men are betaking themselves to their own resources. Mouldy, rusty, rotten theories, that have been thrust upon the mind as golden truths, are cast aside, and truth, with avidity, is embraced because it is seen.

Theories are not now received simply because they have been sanctioned by time and custom; but, in order now to be received, must bear a rigid investigation under a full flow of light. In this age of the world men must think for themselves upon all subjects. It is impossible for us to be blind to a change so great and so apparent, and which progresses so rapidly toward an entire revolution. This mental servility is numbered among the things that are to pass away.

The progressive spirit of the age is even now bearing it along, as on life's rapid current to oblivion. All attempts to check it in its onward course have failed in their aims. The mandates of kings and councils cannot restrain that freedom of thought which is everywhere bursting forth; even when the most cruel despotism has been exercised for its suppression, it comes gurgling up from the inmost depths of the human soul. It can no longer be dissipated by the power of frowns or smiles. Is there any probability that the mind will settle back to its former bondage? The condition and tendencies of all things preclude the possibility of such a return. Backward it cannot go,—the land of promise is before it, and onward must be its career.

At all this, however, modern conservatism stands horror-stricken, sees nothing but evil; the world turned up side down; all things out of joint, and going to ruin.

> "Down deep in a hollow some wiseacres sit,
> Like the toad in his cell in the stone,
> Around them, in daylight, the blind owlets flit,
> And their creeds are by ivy o'er grown.
> The streams may go dry, and the wheels cease to ply,
> And their glimpses be few of the sun and the sky,
> Still they hug to their breasts every time-honored guest,
> And slumber, and doze, in inglorious rest.
> For no progress they find in the wide sphere of mind,
> And the world's standing still with all of their kind
> Contented to dwell deep down in the well,
> Or move, like the snail, in the crust of his shell,
> Or live like the toad in his narrow abode,
> With their souls closely wedged in a thick wall of stone,
> By the grey weeds of prejudice rankly o'er grown."

Great activity is another distinctive feature of this age. This is the living spirit in the wheels of progress; by it all that is great and glorious has been achieved. Almost all subjects call forth this activity. What prodigious enterprises have been set on foot and carried forward with incredible rapidity, under the influence of a spirit of accumulation of wealth. It has increased with a desire for gain. By this passion genius and skill have multiplied labor an hundred fold, in the application of the arts and sciences to almost every department of industry. Through it commerce is girdling the earth with iron bands, and connecting every great centre of trade throughout the world with the electric nerve of the telegraph. Rapidity of action is universally sought, and all things are esteemed in proportion as they possess this quality. Influenced by this feeling, men take the fastest train, and then complain that it goes too slow. They send their messages by lightning rather than by some more earthly conveyance. The fleetest ship is most highly prized; and the most rapid thought is alone deemed worthy of record.

In almost every movement of the age, the first question is, "How quick can it be done?" and the next, "What is it worth?"

*A distinguishing feature of this age is its utilitarian character;* a disposition to turn every thing to the best account. Though a large void yet remains to be filled, the debtor side of the page shows a heavy balance against us, it is encouraging to consider that some progress is being made. The history of the world, so far as it has come to us, is but a record of the fact that the disposition of its wealth, talents and energies has been, not only useless, but pernicious in its tendency. The principle part of the world's written history, gives us little else than the fact that men have, from the beginning, been prodigals, spendthrifts and abusers of every gift that God ever gave them. Nineteen-twentieths of all earth's treasure, over which man has had control, has been prostituted to base purposes; aye, more, it has been brought, offered, and laid upon the altar, a willing sacrifice to the god of desolation, ruin and death.

Uusually, in proportion as a man possessed wealth, he was a curse to his race. For it was not only hoarded, and prevented from accomplishing errands of blessing and mercy to man, but it was made a sharp and bitter curse, and used as a double edged sword against his fellows.

How have men in the by-gone ages of the world employed the powers of mind? That external principle upon which was stamped in the beginning the daguerreotype of the Mighty Maker of the Universe; that principle that has its beginning in this world and its termination nowhere, that lives with increasing vigor and energy through myriads of ages.

The intellect of man has been cultivated for many and various purposes. Many have received no cultivation at all except that communicated by observation, and that, too, in a very limited sphere. In some, the baser qualities only have been cultivated. In other cases, all the faculties are cultivated, but only to be prostituted to ignoble purposes.

One grand object for which men in all ages of the world have employed the intellect is the procurement of wealth. Some have been thus employed solely for the sake of the possession; such is the miser, who is never satisfied with increase, but his desire grows with his accumulations; and like the remorseless jaws of death, he never says it is enough. He drinks insatiately that which makes him more thirsty; and continually adds fuel to that fire which burns out his vitals. Such ever has been, and is the love of money. Others have sought wealth that they may consume it for the gratification of the appetites and lusts. The case of the spendthrift is less deplorable than that of the miser; for while the victim is no more certainly destroyed by the former than the latter propensity, the prodigal distributes to others that which he has obtained, and it may do good elsewhere. We have an instance of the return of a prodigal to a path of uprightness, but in no instance the return of a miser.

Others have given all the energies of the mind to procure wealth, because it gives them power and influence over their fellow men. The minds of men have been cultivated with a view to the gratification of ambition,—emulous for fame and power. But those who have done most in this respect, have fared the worst. The names of those who built the pyramids of Egypt, through a vain desire for renown, have become as deeply wrapped in oblivion as those of their meanest slaves. Others of a more recent date, have, however, by the enormity of their crimes, perpetrated with a view to render their names immortal, succeeded in handing them down to posterity. But, oh! what fame! Names rolled in blood. A change in all these respects is now going on. The wealth of the world is better appropriated now than ever before. It is bestowed upon objects in proportion as they are useful to mankind.

The arts and sciences are valued and appreciated as they are susceptible of application to useful purposes. Other ages have doubtless possessed operations and processes of which we are ignorant; for this, however, we have no regret. Of these a great proportion required a vast expenditure of time, labor and money, without a corresponding utility; and it is highly probable that if a knowledge of them was yet retained, their use would be abandoned in this utilitarian age. Who would now think of spending twenty or thirty years in ornamenting a charnel house?

The ancients made Corinthian Brass. We have hundreds of different alloys applicable to a thousand useful purposes, unknown to them. They made Damascus blades. We have the metals wrought into ten thousand useful forms of which they never conceived. They made glass malleable. We convert it into a thousand different useful forms unknown to them.

Men are appropriating the powers of mind to accomplish good for mankind. Many great intellects are wholly devoted to the amelioration of the condition of humanity. The number thus devoting themselves is increasing. The uprising generation is educated not with a view to wealth, nor fame, but that they may be benefactors to mankind. By the proper application of wealth, talent and energy, the car of civilization and religion is made to roll in glory and majesty over the world, bearing its precious freight of truth, liberty and freedom from the galling bondage of ignorance and superstition.

GENTLEMEN GRADUATES:—By a consideration of the facts presented, you can perceive, to some extent, the position you should occupy as men. It is manhood in its integrity, in its purity of motvie and holiness of action, that is first desirable. With it you are fitted for any position in which circumstances may place you; without it you are unworthy of any honorable position. and unable to command the confidence of any. Other attainments cannot compensate for a want of this quality. All things about you are undergoing change; revolution succeeds revolution. Your lot is cast in an eventful period of the world. Old theories and practices are overturned, and succeeded by new ones; new principles are continually arising that require the closest scrutiny. In this conflicting state of things you are necessarily thrown upon your own resources. And we doubt not, you will acquit yourselves with honor, and with the integrity of true men. Always place yourselves upon the side of progress. And in regard to the profession you are pursuing so eagerly, let industry, perseverance and integrity of manhood accompany you.

If in the exercise of your professional duties, the good of humanity is not a prominent idea; abandon it.

Gentlemen.—You have placed yourselves in a very responsible position; your profession is one intimately connected with the welfare and comfort of your fellow men. It is yet in its infancy; in its developing stage: and it rests with you whether it shall be a full development, perfect and symmetrical in all its parts; or whether it shall be a meager, withered starveling. You must not only exercise a sustaining influence, but it must be made to grow and expand to full dimensions. But do you ask me "how this is to be done?"

First draw upon your own resources, your industry, your perseverance, your talents, your skill and genius. But do not stop here. Reach out into all the fields of science, and wherever you find anything capable of assimilation and appropriation take it. Make all the arts and sciences pay tribute to your profession. Extend the arm of investigation; select and gather in all that you can use. Turn every thing to the best account; conform to the utilitarian spirit of the age.

Lately our pupils, you are now in every sense our professional brethren. We welcome you, and rejoice in the prospect of your aid as co-laborers. But the time has come that we must part. Go forth with our best wishes; In the name of the faculty I bid you *Farewell.*

The following response was made in behalf of the class, by Dr. C. H. James, one of the graduates:

*Gentlemen of the Faculty of the Ohio College of Dental Surgery:*—

It is with emotions of pain and pleasure that I rise to perform this, the last duty of this occasion. With emotions of pain, because the connection which has existed between us for so short a time is about to be broken by our separation, having attained the ultimatum of this institution. With feelings of pleasure, because we are about to take a position in that profession which will be an honor to us all, and which we will ever strive to sustain and place upon a footing which will rival that of law or medicine, if that point has not already been attained. Be assured, gentlemen, that Excelsior will be our motto, and with it upon our banners, we will ever strive to guide our noble ship beyond the shoals of prejudice, to outride the storms and tempests which may, and invariably will, cross our path. We do not, and indeed cannot, too deeply appreciate the care with which you have pointed out the landmarks of this science,—a science which has been much abused by falling into the hands of the ignorant and unskilled. Much might be said upon this latter division, but we cannot at this time give it its merited attention. The unskilled blacksmith and more barbarous barber, have been supplanted by the educated and skillful professor. But to accomplish this, what has been the extent of your labor and the difficulties with which you have been beset? So with medicine. Where now is the medicine-man, so called on account of his pretensions, and the old woman of roots and witchcraft? Go visit the halls of our colleges and notice the intellect and hoary locks of those who have thrown aloft their standards and planted them upon the highest battlements, having overthrown the bulwarks of ignorance and superstition.

We are glad that we are promoted to the rank which is destined to continue this crusade, and that we will have an opportunity, by proper deportment and study, of meriting our new position. A vast field is before us, and in it we discover bright spots toward which we shape our course. These have long been in view, and without your aid how varied would have been our march? To you we owe it that the most direct course has been pointed out. You have taught us the bearings and placed in our minds, by your teachings and example, a compass which will not vary, and which will, if strictly followed, bring us all safely to the point toward which our ambition leads us. Upon this spot, and at this time, a monument is erected in our memories which will ever be visible to us, wander where we will, or let fate, be it what it may, overtake us. Neither will we strive to efface this monument from our memories, but will make it the focus of our energies, and when we part hence, we will not do it with an idea of never seeing or visiting it more, or of disclaiming all connection therewith, but will look to the privilege of returning and greeting you all again, as ours, and will flatter ourselves that we will meet you with outstretched hands and a ready welcome on your lips.

This is a commencement not only of our labors but also of our studies. Our books are but fairly opened, and our minds fixed upon the contents. Your light is reflected upon the pages, and every thing is made plain and easy.

We would like to dwell longer upon this subject, but will delay but a moment. With this scepter* as a token of our attainments, we hope to conquer every difficulty in our paths, and with this *Holy Light*† we have pointed out to us a bright future even beyond the grave. It will be our pleasure, as it is our privilege, ever to follow its teachings; and in its pages, when disease is wasting our frames, and our final dissolution is at

* The Diploma. | † The Bible.

hand, may we find that our anchor of hope has been cast in a haven of everlasting peace. And now, gentlemen, may you be spared to see your most sanguine expectations in us and our progress realized, and may you never have cause to regret having entrusted us with a treasure which is as dear to you as it is of importance to the whole human race.

